# Zishen Huoxue Recipe Protecting Mitochondrial Function of Hypoxic/Reoxygenated Myocardial Cells through mTORC1 Signaling Pathway

**DOI:** 10.1155/2020/8327307

**Published:** 2020-07-27

**Authors:** Ruxiu Liu, Xing Chang, Jie Li, Yao Shunyu

**Affiliations:** Guang'anmen Hospital, Chinese Academy of Traditional Chinese Medicine, Beijing 100053, China

## Abstract

**Objective:**

This study focuses on the role of Zishen Huoxue Decoction (ZSHX) in reducing mitochondrial membrane potential and reducing the proportion of apoptosis through the mTORC1 signaling pathway.

**Methods:**

In our experiment, we first constructed an in vitro hypoxia/reoxygenation (H/R) model of H9C2 cells. Then, the cells were divided into control group, model group (hypoxia/reoxygenation, H/R), ZSHX, ZSHX + Rapa, low-dose ZSHX (100 *μ*g/ml), and middle-dose ZSHX. High-dose ZSHX (400 *μ*g/ml) group was treated with Zishen Huoxue Decoction (ZSHX). Western Blot was used to detect the expression of cell-related protein and RT-PCR was used to detect the expression of the cell-related gene in each group. Flow cytometry was used to assay for ROS content and the apoptotic ratio of H9C2 cells, Seahorse Live Cell Energy Meter was used to detect the Mitochondrial Respiratory Function in H9C2 Cells, and confocal laser scanning was used to detect the mitochondrial membrane potential of H9C2 cells.

**Results:**

Western Blot assay showed that the relative expression of mTOR and Raptor in the H/R group was significantly lower than that in the control group (*n* = 3, *P* < 0.05). The expression of mTOR and Raptor was upregulated and the relative expression of 4E-BP1 was downregulated in the middle- and high-dose ZSHX groups (*n* = 3, *P* < 0.05). In addition, the ROS content of H9C2 cells was detected by flow cytometry, showing the ROS synthesis in H/R group (78.31 + 6.14) higher than that in the control group (34.53 + 6.10) (*n* = 3, *P* < 0.01). The ROS value was increased significantly after rapamycin inhibited mTOR (66.18 (+4.03 vs. 52.31 (+6.01), *n* = 3, *P* < 0.05). The basal mitochondrial respiration and ATP production in H/R group were significantly lower than those in the control group (38.17 + 17.76); the mitochondrial leakage in H/R model group was significantly higher than that in the control group (H/R: 40.93 + 5.18 vs. Ctrl: 27.17 + 8.92, *n* = 4, *P* < 0.05). The apoptotic rate of cardiomyocytes in the H/R model group (70.91 + 4.57) was significantly higher than that in the control group (14.52 + 2.37, *n* = 3, *P* < 0.01), and Zishen Huoxue Decoction could decrease the apoptotic rate of hypoxic-reoxygenated cardiomyocytes (ZSHX: 18.24 + 4.17 vs. H/R: 78.91 + 3.48, *n* = 3, *P* < 0.01).

**Conclusion:**

ZSHX Decoction has the effects of activating mTORC1, inhibiting the overexpression of 4E-BP1, inhibiting fatty acid oxidation, protecting the respiratory function of mitochondria, reducing ROS and apoptosis, and thus protecting myocardial cells from injury.

## 1. Introduction

Ischemic heart disease is currently the key cause of disease mortality worldwide [1]. In recent years, many studies have elucidated the intrinsic adaptive mechanism of myocardial ischemia and reperfusion injury and revealed the complex pathological changes caused by myocardial ischemia and reperfusion injury, including the destruction of cell energy, ion homeostasis, and oxidative stress. Studies have also confirmed that these pathological changes are concentrated on mitochondrial dysfunction [2, 3]. Mitochondria are important energy-supplying organelles in cells, playing an important role in the survival of myocardial cells and the maintenance of normal cardiac function. What's more, studies have confirmed that myocardial contractility and cell homeostasis are almost entirely dominated by adenosine triphosphate (ATP) produced by mitochondria. ATP is produced by oxidative phosphorylation of a series of subunit complexes embedded in the mitochondrial inner membrane as an electron transfer chain (ETC). The potential energy released during electron transfer drives protons to be pumped out from the mitochondrial matrix side to the mitochondrial inner membrane and then forms an electrochemical gradient, i.e., mitochondrial membrane potential (MMP). Current studies have shown that I/R can lead to a decrease of mitochondrial respiratory chain enzyme activity, mitochondrial membrane potential, cell apoptosis, and so on. The direct intervention of mitochondrial respiratory chain or indirect regulation of mitochondrial membrane potential is considered to be an important method to prevent and treat myocardial ischemia-reperfusion injury [4]. Therefore, this paper mainly discusses the main mechanism of Zishen Huoxue Decoction in regulating mitochondrial membrane potential and protecting mitochondrial function.

Zishen Huoxue Decoction is an effective original prescription for the treatment of patients with coronary heart disease and after percutaneous transluminal coronary intervention. The main components are mulberry, *Trichosanthes*, onion, *Panax notoginseng*, and Radix Pseudostellariae. Our team has systematically reported the clinical observation of Zishen Huoxue Decoction on patients with coronary heart disease and after PCI, and the results showed that the prescription could significantly improve the clinical symptoms of myocardial ischemia such as chest tightness, chest pain, and shortness of breath [5]. It has the effects of alleviating angina pectoris, reducing the dosage of nitroglycerin, and improving the changes of ST-T in ECG ischemia and has no effect on liver and kidney function and routine blood and urine, which is safe and effective in clinical medication [6]. At the same time, Zishen Huoxue Decoction can also reduce the levels of blood lipid-related indicators Hcy, MMP-9, FIB, and ET, which indicates that it has the effects of regulating lipid metabolism, stabilizing plaque, anti-inflammation, and antithrombosis, and protecting vascular endothelial function [7]. Basic experimental studies found that this recipe can also improve the pathological morphology of ischemic myocardial fibrous swelling and deformation, tissue loosening, and edema, reduce ET, IL-18, and p38MAPK, increase Hsp27, and play an anti-inflammatory, stable plaque, antioxidant, and antiapoptotic role. At the same time, proteomic studies showed that this recipe could downregulate LDH-B, CPT-1, and IDH, upregulate ATP alpha and MYH, improve energy metabolism of myocardial cells, and protect the cytoskeleton of ischemic rats [8]. It is suggested that Zishen Huoxue Decoction can regulate the energy metabolism of myocardial cells and protect the cytoskeleton [9, 10] by upregulating ATP synthase alpha and downregulating CPT-1. However, the effect of Zishen Huoxue Decoction on mitochondrial energy metabolism and its specific mechanism are still unclear.

Mammalian target of rapamycin (mTOR) is a key factor involved in the control of cell nutritional induction and growth. The mTOR complex 1 (mTORC1) can regulate protein synthesis, cell growth and proliferation, cell autophagy, cell metabolism, and stress response. MTORC1 mainly contains three main components: mTOR, Raptor, and mLST8, of which Raptor is the key regulatory and structural subunit of mTORC1. The human body regulates the energy metabolism of myocardial cells mainly through the mTOR-Raptor complex. Many studies have shown that mTORC1 is closely related to I/R. It can protect I/R injury by influencing the mechanisms of the mitochondrial respiratory chain, opening mitochondrial permeability transition pore, and upregulating antioxidant genes and autophagy level. The overexpression of mTORC1 in myocardial cells can reduce cardiac remodeling induced by I/R. Dowling team [11, 12] proposed the concept of the mTORC1/4E-BP regulation axis in science; that is, energy metabolism disorder regulates eukaryotic translation initiation factor 4E-binding proteins (4E-BP) through mTORC1, which causes a series of downstream molecular functional changes and induces pathological state. 4E-BP is the main regulator of mTORC1 affecting mitochondrial biosynthesis and function. The existence of the regulatory axis and its mechanism has been verified by other scholars. Inhibition of mTORC1 can decrease ATP synthase level and affect mitochondrial DNA content, mitochondrial mass, and cell ATP level. However, in 4E-BP deficient cells, the inhibition of mTORC1 on ATP synthase is not obvious, suggesting that 4E-BP deficiency may promote the expression of mTORC1 and ATP synthesis. Many studies have shown that traditional Chinese medicine can improve the energy metabolism of ischemic myocardium by regulating the activity of mitochondrial respiratory enzyme complex and transmembrane potential of myocardial cells [13–15]. In order to better elucidate the pathological mechanism of I/R and the specific mechanism of Zishen Huoxue Decoction in protecting ischemia/reperfusion, we used H9C2 cells to simulate hypoxia/reoxygenation model in vitro and used Western Blot, RT-PCR technology, and key molecular inhibitors of mTORC1 pathway to intervene to explore the protective effects of mTORC1 signaling pathway and 4E-BP regulation axis on hypoxia/reoxygenation. Therefore, the mechanism of mitochondrial function of oxygen myocardial cells was studied, and the effects of different concentrations of Zishen Huoxue Decoction on mitochondrial function of hypoxia/reoxygenation myocardial cells were examined by Seahorse energy measurement and laser confocal technique.

## 2. Materials and Methods

### 2.1. Experimental Animals

Healthy male Wistar rats were purchased from Beijing Sparford Biotechnology Co., Ltd., and H9C2 rat embryonic cardiomyocytes were purchased from Cell Resource Center, IBMS, CAMS/PUMC, Institute of Basic Medicine, and Chinese Academy of Medical Sciences.

### 2.2. Experimental Materials

Dimethyl sulfoxide and fetal bovine serum (FBS) was purchased from Gibco Company. DMEM high sugar medium, trypsin, penicillin, streptomycin, phosphate buffer solution, DAPI, polyformaldehyde Mitotracker XF cell mitochondrial oxygen consumption detection kit, 1X PBS buffer, mitochondrial membrane potential detection kit, Annexin V-FI TC cell apoptosis kit, 5 × loading buffer, NC membrane, a protein phosphatase inhibitor, 4 × loading buffer, SDS-PAGE kit, 30% polyacrylamide, 1 M Tris-HCI (PH 6.8), 1.5 M Tris-HCL (PH 8.8), 10% SDS, 10% ammonium persulfate (APS), and ECL Plus hypersensitive luminescent solution were purchased from Trizol Reagent from Beijing Soleboard Technology Co., Ltd. Glutamine was purchased from Gibco Company and Sodium Pyruvate from Amresco Company. Rapamycin, anti-mTOR antibody, anti-Raptor antibody, anti-p4E-BP1 (Ser65) anti-P70s6k antibody, anti-CPT1A anti-ATP5D antibody (Mice), anti-GAPDH antibody, Shan Sheep anti-rabbit IgG/HRP, and rabbit anti-mouse IgG/HRP were purchased from Abcam.

### 2.3. Recovery and Culture of H9C2 Cells

H9C2 rat embryonic cardiomyocyte line was used in the experiment. H9C2 cells were taken out of liquid nitrogen and melted in a water bath at 37°C (within 1 min). The cryopreservation solution was removed. The cells were placed in a sterile centrifugal tube and centrifuged (1380 r/min, for 5 min). After centrifugation, the H9C2 cells were cultured in 3 ml complete medium (containing 10% fetal bovine serum, 100 k*μ*/L penicillin, 100 mg/L streptomycin, and DMEM high sugar medium). After 24 h, the cells were replaced with 4 ml medium and then cultured in the incubator.

### 2.4. Generation and Cryopreservation of H9C2 Cells

Cell generation: cells were placed in an incubator at 37°C and 5% CO_2_. When 80%–90% of the cells were fused, the old culture medium was discarded, PBS was cleaned, and 0.25% trypsin was digested in the incubator for 1 min. Cell morphology was observed under a microscope, then digestion was terminated by adding DMEM, and centrifugation was performed at 1380 r/min for 5 min. Cell cryopreservation: continue to pass on the cells (4–6 times); if the cells are in good condition, they will be cryopreserved. The specific methods of cryopreservation are as follows: (1) remove DMEM, wash cells 1-2 times with PBS, then add 0.25% trypsin of 1 ml, digest cell dishes in the incubator for 1 min, quickly get them to the microscope to observe the cell morphology, and then add DMEML to stop digestion. The cells were transferred to a 15 ml centrifugal tube, centrifuged (1380 r/min, for 5 min), and then the cryopreservation solution was poured into the cryopreservation tube. Put it in 4°C refrigerator for 10 min, −20°C refrigerator for 90 min, and−80°C overnight, and finally put it in liquid nitrogen.

### 2.5. Preparation of Serum Containing Drugs

Referring to our previous experimental study [16], 20 healthy male Wistar rats, half male and half female, weighing 380–400 g, were selected. After a week of adaptive feeding, rats were randomly divided into two groups (blank control group and Zishen Huoxue Recipe group). Adults need 129 g of Zishen Huoxue Recipe, which is equivalent to 4.0635 g of raw medicine/kg in rats. Therefore, rats need 20.3175 g of raw medicine/kg daily, and blank serum group is given the same amount of distilled water. Each g/kg dosage group (equivalent to 5 times clinical dosage) was given orally twice a day (one time in the morning and the other in the afternoon). After 7 days of continuous administration, fasting water was given 12 h before the last administration. The rats were anesthetized with 3% pentobarbital. The rat was placed in a supine position on a flat plate, its limbs were fixed with rope, its abdomen was wiped with alcohol cotton ball, its abdominal cavity was opened with surgical scissors, its viscera was opened, the dorsal medial part of the rat was exposed, and a layer of the thin film was separated with tweezers. As a result, the abdominal aorta on the side of the spinal column could be found, and the inferior vena cava of the rat was dark red; the color of the abdominal aorta is white, and the fascia tissue near the abdominal aorta of uncle is gently peeled off with small tweezers. After the inferior vena cava is separated, the blood collection needle is selected to extract the artery blood and injected it into the serum tube. Then, the serum tube was naturally placed for 2 h to separate the serum. The centrifuge was adjusted to 2500 r/min and then centrifuged for 25 min. Then, the serum was inactivated for 30 min in a water bath at 56°C. Then, the serum was filtered with a 0.22 *μ*m microporous membrane to remove bacteria. Finally, the sterile blank serum and the medicated serum containing the Zishen Huoxue Recipe were obtained, and the serum was separated. Each EP tube was installed and finally put in −80°C refrigerator for freezing.

### 2.6. Preparation and Grouping of the Hypoxia/Reoxygenation Cardiomyocyte Model

Referring to the literature [17], when H9C2 cells adhered to the wall, the old culture medium would be replaced by a pre-N_2_ saturated hypoxic medium. The sealed box was filled with a mixture of about 1 L gas (95% N_2_–5% CO_2_) with a volume of 30 times, so as to fully remove the oxygen left in the prepared sealed box, and then clamped with hemostatic forceps after 5 min. In order to create hypoxic conditions, the intake and outlet pipes were placed in incubators for 2, 4, 6, and 12 hours, respectively, then the high sugar medium needed for cell replacement was taken out, and then the ventilated pipes were opened to restore the oxygen and sugar supply for 16 h, so as to prepare the cell injury model. Groups were divided into control group, model group (hypoxia/reoxygenation, H/R), ZSHX, ZSHX + Rapa, low-dose ZSHX (100 *μ*g/ml), and middle-dose ZSHX (200 *μ*g/ml). High-dose ZSHX (400 *μ*g/ml) group was treated with Zishen Huoxue Recipe.

### 2.7. Changes of 4E-BP1, p70S6K, mTORC1, and Raptor Detected by Western Blot

Western Blot was used to detect the protein expression of 4E-BP1, p70S6K, mTORC1, and Raptor. The total proteins of 4E-BP1, p70S6K, mTORC1, and Raptor could be extracted from cells. The BCA method was used to determine protein concentration. RIPA lysate was used to dilute and adjust the sample concentration and prepare electrophoretic samples. The protein samples were separated in 8% and 12% SDS-PAGE coagulation. The gel was transferred to the nitrocellulose membrane. The protein on the gel was transferred and the polyvinylidene fluoride (PVDF) membrane was used as the base material. The skim milk powder or 5% BSA was added to the antibody incubating box and sealed, shaking the table (40 rpm). The 2H was sealed at room temperature. The antibodies were prepared and incubated and analyzed by grayscale scanning.

### 2.8. Detection of mTOR, Raptor, and 4E-BP1 by RT-PCR

RNA was extracted and the absorbance of extracted RNA was measured by NanoDrop2000 spectrophotometer. The concentration of RNA was measured by absorbance. Reverse transcription was used to synthesize and quantify the DNA. The SYBR Green I kit was used to add 10 *μ*l 2 × SuperReal PreMix Plus, 2 ml 50× Reference Dye, 5.8 ml RNase-free water, 1 *μ*l DNA template, 10 micromol/L forward primer, and 10 micromol/L reverse primer, each of 0.6 ml, to establish a 20 microl reaction system. The primer design is shown in Table 1.

### 2.9. Detection of ROS Content in H9C2 Cells by Flow Cytometry

H9C2 cells were planted in a culture dish with a density of 1*∗*10^6^/mL. The model was the same as those in “2.6.” H9C2 cells were divided into the Ctrl group, H/R group, Rapa + ZSHX group, and ZXHX group. DCFH-DA of 10 UL was diluted according to the ratio of 1 : 1000 and working fluid was allocated. The cell culture medium was removed and washed twice with PBS. Centrifugal cells were digested by the routine method. Cells were collected and added to serum-free medium 1 ml (10 *μ*mo/L DCFH-DA). Cells were incubated in an incubator for 20 min and mixed upside down every 3–5 min. After PBS was washed, 1 ml of serum-free medium was added to suspend the cells and tested on the computer.

### 2.10. Detection of Apoptosis in H9C2 Cells by Flow Cytometry

H9C2 cells were planted in a Petri dish at a density of 1*∗*10^6^/mL. The administration and modeling of H9C2 cells were the same as those in “1.3.” Cells were digested by passage, and DMEM terminated digestion. H9C2 cells were collected after centrifugation for 5 min at 1380 r/min in a 15 ml centrifuge tube. It was added to the flow tube and 100 mL Binding Buffer was added. Finally, 5 *μ*L Annexin V-FITC and 5 *μ*L PI were added to each tube, heated at 55°C for 5 min, and stained at room temperature for 15 min. The apoptosis of H9C2 cells was detected by flow cytometry after adding 400 *μ*L Binding Buffer before boarding.

### 2.11. Detection of Mitochondrial Respiratory Function in H9C2 Cells by Using a Seahorse Living Cell Energy Analyzer

H9C2 cells were treated with 5000–40000 cells/holes, a total of 80 *μ*/holes. Seed cells were placed in the cell plate, and the administration and modeling were the same as those in Section 2.6. Ensure that the background pore is acellular (A1, A12, H1, H12), 80 *μ*l medium was inoculated, and the cells spent the night in the cell incubator. At the same time, the Seahorse instrument and computer were opened, and Seahorse related software was opened, so that the instrument warms up to 37°C, preheating overnight. Then, the XF96 probe plate was taken, 200 UL standard solution was added to each hole, and the upper plate was put in place to ensure that the probe was immersed in the Seahorse XF calibration solution. The test plate was put back on Utility Plate, and the probe was hydrated for 24 h in a 37°C incubator without CO_2_. Seahorse XF Base Medium was used to configure the detection solution. The pH value was adjusted to 7.4 with NaOH, filtered with a 0.22 *μ*m microporous membrane, and incubated at 37°C. The cell plate was then removed and the cell status was observed under a microscope. The growth medium was replaced by a detection medium, the cells were washed twice with detection medium, and 175 *μ*L was retained at last. The incubator was incubated at 37°C for 1 h. Before detection, the final concentrations of 1 micromol/L oligomycin, 1 micromol/L FCCP, and 0.5 micromols/L R/A were added to the probe plate. After 20 min, the cell plate was replaced, and the data were detected on the computer and analyzed comprehensively by Wave software.

### 2.12. Laser Confocal Detection of Mitochondrial Fluorescence in H9C2 Cells

H9C2 cells were seeded in a confocal laser dish, and the dosage and modeling were the same as those in Section 2.6. Mitotracker needs to be preheated at 37°C before use. A working fluid of 1 L is added to the 5 L medium. The cell culture medium is removed. The preheated working fluid (4 ml medium + 1 L Mitotracker) is added to the cell incubator at 37°C for 30 min. The working fluid is removed, the fresh culture medium preheated at 37°C is added, and centrifugation at 1500 r/min is carried out for 5 min. Cells were collected for 3 min, washed with cold PBS buffer, fixed with 4% paraformaldehyde for 10 min, and continued to wash with cold PBS buffer for 3 times. DAPI was added for 5 min, then the cells were washed with cold PBS buffer 3 times, each time for 3 min, and finally, antifluorescence attenuation sealer was added to the machine for observation.

### 2.13. Laser Confocal Detection of Mitochondrial Membrane Potential in Myocardial Cells

H9C2 cells were seeded in a confocal laser dish, and the dosage and modeling were the same as those in Section 2.6. The formula of JC-1 dyeing working fluid is 25 liters JC-1 (200X) + 4 ml ultrapure water + 1 ml JC-1 dyeing buffer. The cells were then washed with PBS, followed by 1 ml of cell culture medium. Then, 1 ml of working fluid was added to the incubator, and then it was fully blended and incubated in the incubator at 37°C for 20 min. X JC-1 dyeing buffer and ice bath were configured. After 20 min, the supernatant of the incubated cells was removed, and the cells were washed twice with the dye solution. Then, 1 ml of cell culture medium was added and placed under laser confocal microscope for observation.

### 2.14. Statistical Method

Data were processed by SPSS21.0 statistical software. Normal distribution measurements were expressed in the form of *X* + *s*, and nonnormal data were tested by the nonparametric test. Paired or two groups of continuous measurement data were analyzed by *t*-test, one-way ANOVA was used for comparison among groups, and SNK-q was used for comparison between groups, of which, *P* < 0.05, representing statistical difference, and *P* < 0.01 for a significant difference.

## 3. Results

### 3.1. Effects of Different Hypoxia/Reoxygenation Conditions on H9C2 Myocardial Cells

**Figure 1:**
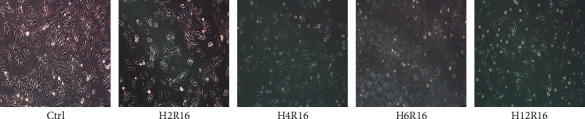
Morphological observation of myocardial cells (200×). H2R16 (hypoxia 2 h/reoxygenation 16 h); H4R16 (hypoxia 4 h/reoxygenation 16 h); H6R16 (hypoxia 6 h/reoxygenation 16 h); H12R16 (hypoxia 12 h/reoxygenation 16 h).

Myocardial cells of H9C2 were hypoxic for 2 h, 4 h, 6 h, and 12 h, respectively, and reoxygenated for 16 h, respectively. Myocardial cells of the control group were cultured under normal conditions. The morphology of H9C2 myocardial cells under different H/R conditions was observed under an inverted microscope, as shown in Figure 1. The results showed that the normal H9C2 cardiomyocytes were spindle-shaped, arranged neatly, uniform in size, with clear cytoplasmic boundaries of the nucleus; however, after hypoxia, with the prolongation of time, the H9C2 cells have shown irregular size in varying degrees, the cell body became round and shriveled, the nucleus enlarged, and there were vacuoles in the cytoplasm, while after hypoxia for 4 h, the morphological changes of H9C2 myocardial cells were observed after 16 h of reoxygenation, especially after 12 h of hypoxia/16 h of reoxygenation.

### 3.2. Zishen Huoxue Recipe on the Expression of mTORC1 Signaling Pathway in Hypoxic/Reoxygenated Cardiomyocytes

**Figure 2:**
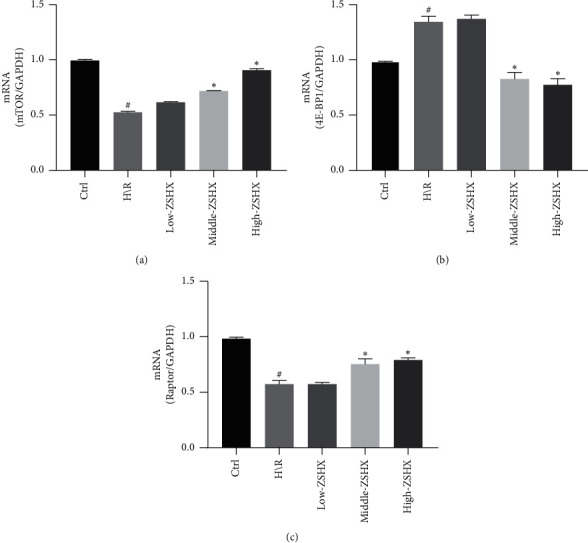
Effects of each group on the expression of mTORC1/4EBP1 signaling pathway in H/R cardiomyocytes. (a) mTOR gene expression map; (b) 4EBP1 gene expression; (c) Raptor gene expression. Compared with the Ctrl group, ^#^*P* < 0.05, and compared with the H/R group, ^*∗*^*P* < 0.05.

In order to explore the molecular mechanism of Zishen Huoxue Recipe in protecting H/R mitochondrial function, we determined the expression of mTORC1 complex (mTOR, Raptor) and downstream molecule (4E-BP1) in the control group, H/R group, and administration group, respectively, and the expression of each group was standardized. According to the control group, the relative expression of mTOR and Raptor in the H/R group decreased significantly, while the relative expression of 4E-BP1 increased (*n* = 3, *P* < 0.05), as shown in Figure 2. Compared with H/R group, the relative expression of mTOR, Raptor, and 4E-BP1 in the low-dose ZSHX group did not change. The expression of mTOR and Raptor was upregulated and the relative expression of 4E-BP1 was downregulated in the moderate- and high-dose ZSHX group (*n* = 3, *P* < 0.05), as shown in Figure 2. These results suggest that moderate and high doses of Zishen Huoxue Decoction can activate mTORC1 in hypoxic/reoxygenated cardiomyocytes and then inhibit the expression of 4E-BP1.

### 3.3. Zishen Huoxue Recipe on the Molecular Expression of mTORC1 Signaling Pathway in Hypoxic/Reoxygenated Cardiomyocytes

**Figure 3:**
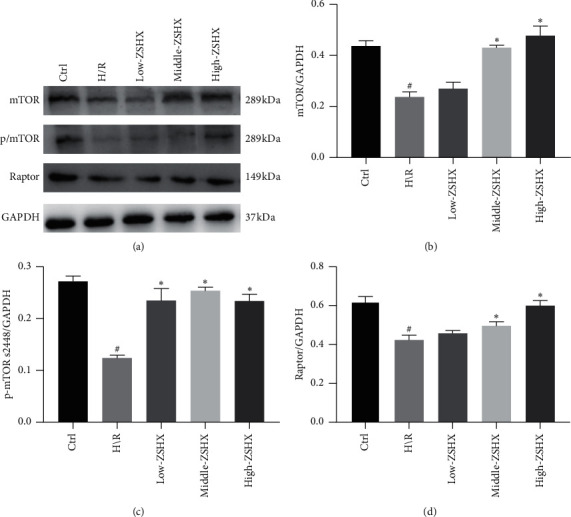
Proteins downstream of the mTORC1 pathway in each group. (a) The expression of macromolecule complexes of mTORC1 and phosphorylated; (b) mTOR protein in each group; (c) p/mTORS2448 protein expression; (d) Raptor protein expression. Compared with the CTL group, ^#^*P* < 0.05, and compared with the H/R group, ^*∗*^*P* < 0.05.

Previously, it was found that Zishen Huoxue Recipe could regulate the expression of the mTORC1/4EBP1 gene, but whether it could regulate the expression of mTORC1/4EBP1 protein needs further confirmation through experimental research. In this part, we determined the expression of mTORC1 complex (mTOR, Raptor) and phosphorylation level (p/mTORS2448) in each group, respectively. The results showed that the levels of mTOR, Raptor, and p/mTORS 2448 protein in the H/R group were lower than those in the control group (*n* = 3, *P* < 0.05), as shown in Figure 3. The expression of mTOR and Raptor protein increased in the middle- and high-dose groups but remained unchanged in the low-dose group. Compared with the H/R group, the phosphorylation level of mTOR (p/mTORS 2448) in each group increased (*n* = 3, *P* < 0.05), as shown in Figure 3. It suggests that Zishen Huoxue Recipe can inhibit the expression of the 4E-BP1 downstream molecule by regulating the mTORC1 pathway in H/R cardiomyocytes.

### 3.4. Zishen Huoxue Recipe on Protein and Gene Expression of Downstream Molecules of mTORC1 Pathway in Hypoxic/Reoxygenated Cardiomyocytes

**Figure 4:**
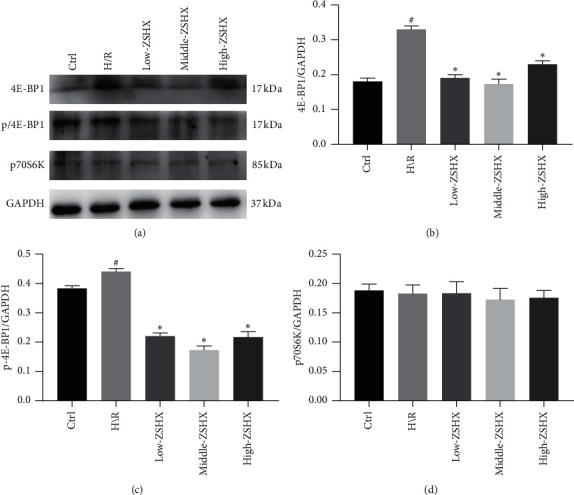
Proteins downstream of the 4E-BP1 pathway in each group. (a) The expression of protein; (b) 4E-BP1 downstream of mTORC1 pathway in each group; (c) p/4E-BP165; (d) p70S6K. Compared with the CTL group, ^#^*P* < 0.05, and compared with the H/R group, ^*∗*^*P* < 0.05.

On this basis, we also explored the effects of Zishen Huoxue Recipe on downstream molecules of mTORC1 pathway, including p70S6K, 4E-BP1, and their phosphorylation levels (p/4E-BP1S65). The expression of 4E-BP1 and p/4E-BP1S65 protein in the H/R group was significantly increased (*n* = 3, *P* < 0.05), while the expression of p70S6K was unchanged, as shown in Figure 4. The expression of 4E-BP1 and p/4E-BP1S65 protein was decreased after H/R model was established with three doses of ZSHX (*n* = 3, *P* < 0.05), as shown in Figure 4. It suggests that Zishen Huoxue Recipe can inhibit the expression of downstream molecules (4E-BP1 and p/4E-BP1S65) by regulating the mTORC1 pathway in H/R cardiomyocytes.

### 3.5. Effects of Zishen Huoxue Recipe Combined with Rapamycin on the Expression of CPT1A and ATP5D in Hypoxic/Reoxygenated Cardiomyocytes

**Figure 5:**
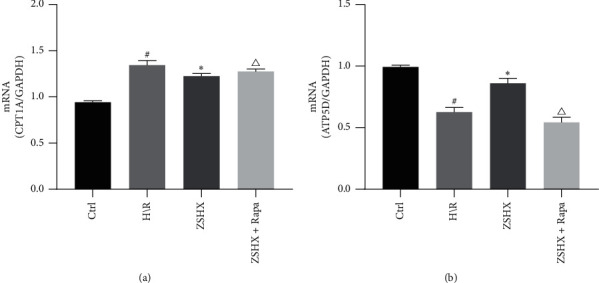
Effects of each group on ATP5D and CPT1A gene expression in H/R myocardial cells. (a) CPT1A gene expression; (b) ATP5D gene expression. Compared with the Ctrl group, ^#^*P* < 0.05, ^*∗*^*P* < 0.05, and compared with the ZSHX group, *P* < 0.05.

Furthermore, we detected the expression of CPT1A and ATP synthase ATP5D in Zishen Huoxue Recipe. The results showed that the relative expression of ATP5D and CPT1A in the H/R group was lower than that in the control group. After the intervention in the ZSHX group, the expression of ATP5D was increased in the H/R group, while the relative expression of CPT1A was decreased (*n* = 3, *P* < 0.05), as shown in Figure 5. Under rapamycin intervention, the expression of ATP5D and CPT1A in the ZSHX group decreased, but the relative expression of CPT1A increased (*n* = 3, *P* < 0.05), as shown in Figure 5.

**Figure 6:**
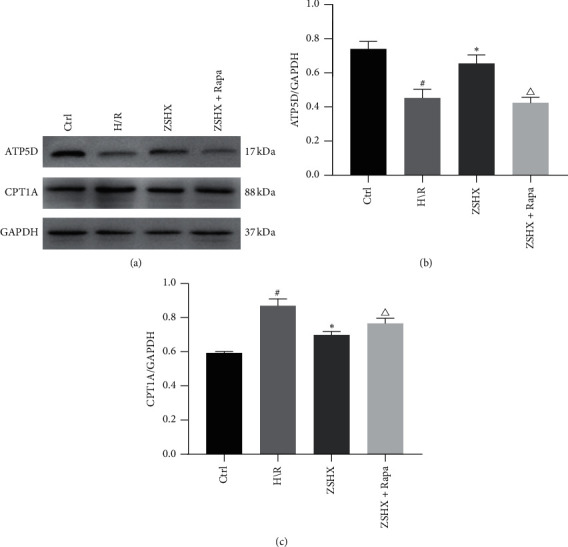
Effects of each group on the expression of ATP5D and CPT1A in H/R myocardial cells. (a) The expression of ATP5D and CPT1A protein in each group; (b) ATP5D protein; (c) CPT1A protein. Compared with the Ctrl group, ^#^*P* < 0.05, ^*∗*^*P* < 0.05, and compared with the ZSHX group, *P* < 0.05.

On this basis, we also detected the expression of PT1A and ATP5D protein in Zishen Huoxue Recipe. Compared with the control group, ATP5D expression was downregulated and CPT1A expression was upregulated in the H/R group (*n* = 3, *P* < 0.05), as shown in Figure 6. The expression of ATP5D was increased and CPT1A was decreased under the action of Zishen Huoxue Recipe (*n* = 3, *P* < 0.05), as shown in Figure 6. After inhibiting mTOR by Rapa, ATP5D value was significantly decreased and CPT1A expression was increased (*n* = 3, *P* < 0.05), as shown in Figure 6 which indicates that Zishen Huoxue Recipe could upregulate ATP5D and downregulate CPT1A expression in H/R cardiomyocytes by regulating mTORC1.

### 3.6. Effects of Zishen Huoxue Recipe Combined with Rapamycin on Mitochondrial Reactive Oxygen Species in Hypoxic/Reoxygenated Cardiomyocytes

**Figure 7:**
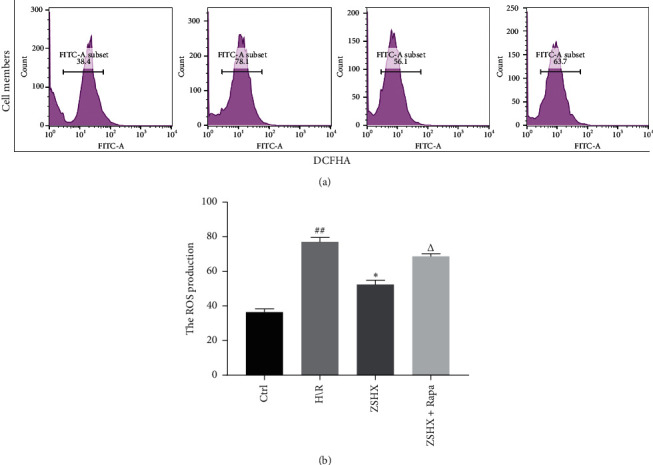
Effects of each group on ROS expression in cardiac myocytes. Compared with the Ctrl group, ^#^*P* < 0.01, compared with the H/R group, ^*∗*^*P* < 0.05, and compared with the ZSHX group, *P* < 0.05.

DCFH-DA was used to label reactive oxygen species (ROS). The effects of Zishen Huoxue Recipe and mTORC1 inhibitor (rapamycin) on ROS production in cardiac myocytes were determined under H/R conditions. The results showed that ROS synthesis in the H/R group (78.31 + 6.14) was higher than that in the control group (34.53 + 6.10) (*n* = 3, *P* < 0.01), as shown in Figure 7. The ROS value of Zishen Huoxue Recipe was significantly lower than that of the H/R group (52.31 (+6.01 vs. 78.31 (+6.14), *n* = 3, *P* < 0.05). Under the action of Zishen Huoxue Recipe, the ROS value was increased significantly after rapamycin inhibited mTOR (66.18 + 4.03 vs. 52.31 + 6.01, *n* = 3, *P* < 0.05), as shown in Figure 7, indicating that Zishen Huoxue Recipe could inhibit the ROS production of hypoxic-reoxygenated cardiomyocytes by regulating mTORC1.

### 3.7. ZishenHuoxue Recipe Combined with Rapamycin on Mitochondrial Respiratory Function of Hypoxic/Reoxygenated Myocardial Cells

**Figure 8:**
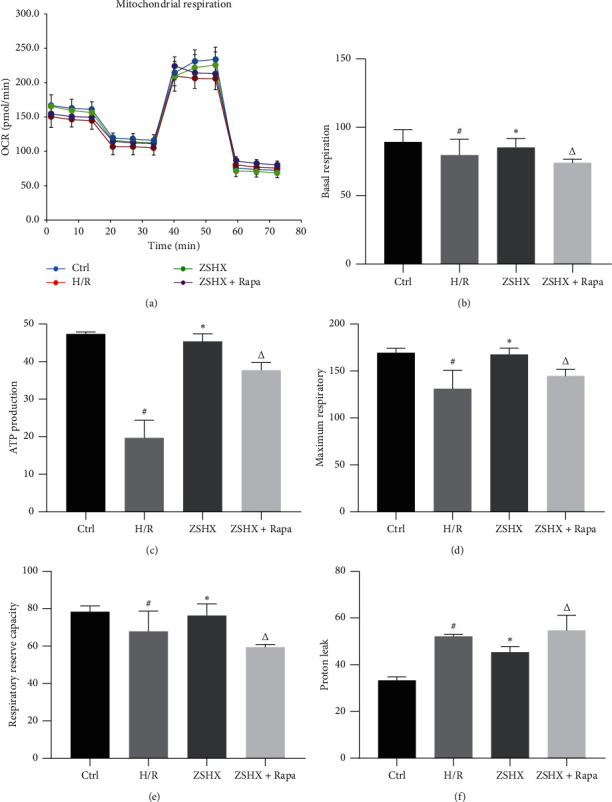
Effects of each group on the expression of mitochondrial respiratory function in myocardial cells. (a) OCR curve; (b) basic respiration; (c) ATP production; (d) maximum respiratory capacity; (e) respiratory reserve capacity; (f) proton leakage. Compared with the Ctrl group, ^#^*P* < 0.05, ^*∗*^*P* < 0.05, and compared with the ZSHX group, *P* < 0.05.

The mitochondrial respiratory curve of the H/R group increased under ZSHX and decreased obviously after adding rapamycin, as shown in Figure 8. Compared with the H/R group, ZSHX increased basal mitochondrial respiration, increased ATP synthesis, decreased proton pump leakage, increased respiratory reserve, and increased maximum respiration (basal respiration: ZSHX: 87.50 + 7.89 vs. H/R: 65.12 + 15.26, *n* = 4, *P* < 0.05; ATP synthesis: ZSHX: 45.10 + 3.99 vs. H/R: 17.12 + 9.76, *n* = 4, *P* < 0.05; proton pump leakage: ZSHX: 42.55 + 5.89 vs. H/R: 57.32 + 2.26, *n* = 4, *P* < 0.05; respiratory reserve: ZSHX: 69.91 + 8.27 vs. H/R: 60.13 + 18.29, *n* = 4, *P* < 0.05; maximum breathing: ZSHX: 155.51 + 18.27 vs. H/R: 125.13 + 25.59, *n* = 4, *P* < 0.05), as shown in Figure 8; When Rapa was added to inhibit mTORC1, the mitochondrial protective effect disappeared and proton pump leakage increased, as shown in Figure 8. The results showed that under the stimulation of Zishen Huoxue Recipe, the basal respiration, respiratory reserve, and ATP production ability of mitochondria increased, which indicated that Zishen Huoxue Recipe could improve the damage of mitochondria caused by hypoxia/reoxygenation, improve proton pump leakage, and increase the production of basal respiration and ATP. When Rapa was added to inhibit mTORC1, the mitochondrial protective effect disappeared and proton pump leakage increased, as shown in Figure 8. It is suggested that the protective effect of Zishen Huoxue Recipe on mitochondria of hypoxic-reoxygenated cells is caused by the mTORC1 pathway.

### 3.8. Effect of Zishen Huoxue Recipe Combined with Rapamycin on Apoptosis of Hypoxic/Reoxygenated Cardiomyocytes

**Figure 9:**
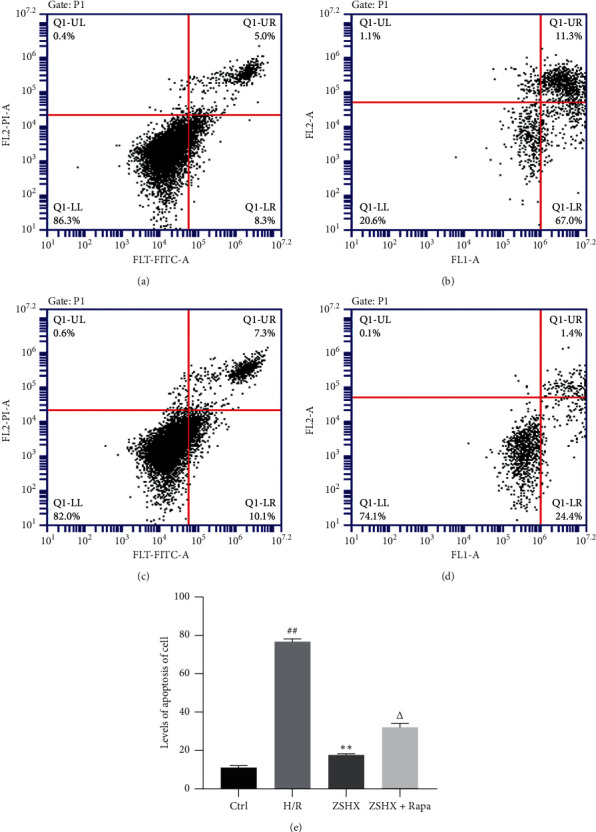
Effects of each group on apoptosis of H/R myocardial cells. (a) Ctrl; (b) H/R; (c) ZSHX group; (d) ZSHX + Rapa group; (e) statistical map of cardiomyocyte apoptosis in each group. Compared with the CTL group, ^#^*P* < 0.01, compared with the H/R group, ^*∗∗*^*P* < 0.01, and compared with the ZSHX group, *P* < 0.05. Compared with the Ctrl group, ^#^*P* < 0.05, and compared with the H/R group, ^*∗*^*P* < 0.05.

The apoptotic rate of cardiomyocytes in the H/R model group (78.91 + 3.48) was significantly higher than that in the control group (13.83 + 3.57) (*n* = 3, *P* < 0.01), as shown in Figure 9. Zishen Huoxue Recipe could decrease the apoptotic rate of hypoxic-reoxygenated cardiomyocytes (ZSHX: 18.24 + 4.17 vs. H/R: 78.91 + 3.48, *n* = 3, *P* < 0.01), as shown in Figure 9. Rapamycin increased the apoptotic rate of the original Zishen Huoxue Recipe group (ZSHX + Rapa: 26.84 + 3.26 vs. ZSHX: 18.24 + 4.17, *n* = 3, *P* < 0.05), as shown in Figure 9, indicating that Zishen Huoxue Recipe could downregulate the apoptotic rate of hypoxic/reoxygenated myocardial cells by regulating mTORC1.

### 3.9. Effect of Zishen Huoxue Recipe on Mitochondrial Membrane Potential of Hypoxic/Reoxygenated Myocardial Cells

**Figure 10:**
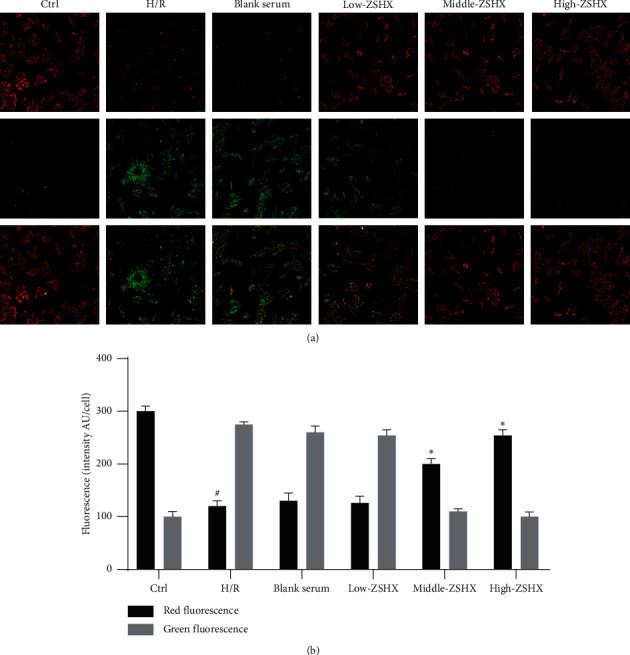
JC-1 was used to detect the mitochondrial membrane potential in each group. (a) Mitochondrial membrane potential fluorescence map; (b) mitochondrial membrane potential fluorescence value. Red fluorescence: J-aggregates green fluorescence: monomer scale is 10 microns. Compared with the Ctrl group, *P* < 0.05, and compared with the H/R group ^*∗*^*P* < 0.05.

In order to further detect the effect of Zishen Huoxue Recipe on the function of H/R mitochondria, the mitochondrial membrane potential was measured. Compared with the control group, the red fluorescence of the H/R group decreased, while the green fluorescence increased (*n* = 3, *P* < 0.05), as shown in Figure 10. The mitochondrial membrane potential (ΔΨ*m*) in the H/R group was significantly higher than that in the control group. The red fluorescence of medium and high concentration of ZSHX increased, while the green fluorescence decreased, suggesting that medium and high concentration of ZSHX can stabilize ΔΨ*m*, while low concentration of ZSHX has no obvious change, as shown in Figure 10. It shows that the prescription of Zishen Huoxue Recipe only has the function of stabilizing ΔΨ*m* and protecting the structure of the mitochondrial membrane above the medium concentration.

## 4. Discussion

This study has first confirmed that the expression of mTORC1 complex and phosphorylation was downregulated in hypoxic/reoxygenated cardiomyocytes, resulting in the overexpression of the downstream molecule 4E-BP1 regulatory axis. In addition, our results show that the main regulatory mechanism of the ZSHX recipe on energy metabolism is through increasing the expression level of the mTOR signaling pathway and lowering the expression level of 4E-BP1, which leads to the increase of basal mitochondrial respiratory level and ATP production. That ZSHX recipe can improve the mitochondrial respiratory function of cardiac myocytes and reduce the apoptotic level of cardiac myocytes by increasing the expression of the mTORC1/4E-BP1 signaling pathway.

The experimental results are consistent with those of Aoyagi and Morita. Aoyagi et al. [18] and other scholars have found that the positive regulation of the mTORC1/4E-BP1 signaling pathway can play a protective role in cardiomyocytes during ischemia-reperfusion. ZSHX recipe can maintain the physiological function of cardiomyocytes by regulating the mTORC1/4E-BP1 signal pathway and then affecting the respiratory function and oxidative stress of cardiomyocytes. Inouye et al. [19] found that in the cells lacking 4E-BP1/2, the decreased expression of the mTOR signaling pathway can increase the protein translation effect of eIF-4E, thus reducing the level of ATP production in cells, leading to mitochondrial function damage. It also indirectly provides a reliable basis for the ZSHX recipe to reduce the protein expression of 4E-BP1 and improve the level of mitochondrial ATP production by activating mTORC1 signal pathway.

Zhou et al. [20] found that when myocardial ischemia occurs, the ion transfer chain in myocardial cells was also destroyed, which would also cause the excessive production of ROS, induced cardiac myocyte apoptosis, and aggravated the reperfusion myocardial injury. On the basis of this study, we also found that the ZSHX recipe can reduce the apoptosis of cardiomyocytes induced by mitochondrial ROS explosion. The effective inhibition of ROS and the protection of mitochondrial respiratory function may be achieved by activating the mTORC1/4E-BP1 signaling pathway.

Samudio et al. [21] found that the CPT1 gene is the rate-limiting enzyme of fatty acid oxidation, and inhibiting CPT1 can reduce the intake of fatty acid into mitochondria, thereby reducing fatty acid peroxidation. The main pathway of lipid consumption is through mitochondrial fatty acid *ß*-oxidation (FAO). The transport of long-chain acyl-coenzyme A ester to the mitochondrial matrix is mediated by the carnitine palmitoyltransferase (CPT) system. CPT1 is considered to be the key regulatory mechanism to maintain fatty acid metabolism balance. Our research results are consistent with those of Samudio et al. [21]. ZSHX recipe can promote aerobic respiration, improve the efficiency of oxygen utilization, and optimize the ratio of respiratory energy, which is conducive to the generation of myocardial energy in the environment of hypoxia/reperfusion and reduces the damage of myocardial cells by downregulating the protein expression of CPT1A. In addition, the inhibition of CPT1A expression by the ZSHX recipe can reduce the peroxidation of fatty acids and further damage myocardial cells caused by oxidative stress.

Different from the results of these studies, Zhu et al. [22] found in animal experimental studies that in adult mice with mTOR induced cardiac damage, fatty acid oxidation decreased significantly, while glucose oxidation increased. However, Cornu et al. [23, 24] reported that mTORC1 can induce oxidative metabolism and mitochondrial biogenesis by promoting the expression of PPAR-*γ* coactivator-1*α* (PGC1*α*). These results support the view that mTORC1 activation can promote fatty acid oxidation. The reasons for these different conclusions are not clear, which may be due to the different roles of mTORC1 in mitochondrial function and oxidative metabolism in different cell types and tissues. ATP5D is an important subunit of ATP synthetase. ATP content is positively correlated with the ATP5D expression level. Most ATP is produced by ATPase located in mitochondria, which consists of two regions and a total of eight subunits, including ATP5D. ATP deficiency affects a series of ATP consumption processes, including cationic pump and F-actin polymerization. Tang et al. [25] recently found that the synthesis disorder of ATP5D may be involved in ATP consumption during ischemia/reperfusion injury, thus presenting an energy metabolism disorder characterized by ATP deficiency. This mechanism was also verified by Tang et al. and other researchers on the mechanism of Qishenyiqi dropping pills [25]. In this study, we found that the ZSHX recipe can also upregulate the expression of ATP5D protein, thus improving the expression level of ATP, alleviating the damage of mitochondrial structure and function, and playing the protective role of cardiomyocytes.

The research of Zhou et al. [9] shows that Qiliqiangxin formula (qlqx) can enhance glycolysis and oxidative metabolism of acute isolated rat cardiomyocytes and increase the number of active mitochondria. By activating PGC-1*α* and mTOR, it can further change mitochondrial energy metabolism [10]. Cao et al. [26] also proved that isoquercetin can improve the activity of H9c2 cells after H/R treatment in terms of protecting mitochondrial function and preventing the leakage of cytochrome C in mitochondria, reducing ROS production and apoptosis. The above research shows that the application of traditional Chinese medicine in myocardial cell protection is also expanding. The prescription of ZishenHuoxue Recipe mainly consists of mulberry, *Trichosanthes*, *Allium macrostemon*, pseudoginseng, ginseng, astragalus, and *Poria*. The protective effect of ZishenHuoxue Recipe on cardiomyocytes may be the therapeutic effect of active components of *Panax notoginseng* saponins, ginsenosides, astragaloside A, and other drugs in the recipe through the regulation of mitochondrial energy metabolism and related signal pathways of cardiomyocytes [20, 27]. Through animal experiments, Liu et al. [28] found that ginsenoside Rb-1 can enhance the activity of antioxidant enzymes and reduce the level of ROS and myocardial oxidative stress injury. Experimental myocardial ischemia and reperfusion injury in rats has a significant protective effect. Huang et al. [29] found that astragaloside can significantly enhance cell viability, inhibit the production of ROS, increase the level of oxidative stress, reduce the loss of mitochondrial membrane potential, and ultimately slow down the process of cardiomyocyte apoptosis. Wang et al. [30] showed that the total saponins of *Panax notoginseng* can increase the levels of serum superoxide dismutase (SOD), reduced glutathione (GSH), and catalase (CAT) and have strong anti-free radical and antioxidative stress effects. The above experimental results at the same time confirmed that the ZSHX recipe can reduce the oxidative stress injury of myocardial mitochondria by regulating ROS, which may be related to ginsenoside, astragaloside, and total saponins of *Panax notoginseng*. The protective effect of the active ingredients in other drugs on cardiomyocytes needs to be further verified.

In summary, we found that hypoxia/reoxygenation inhibited the expression of the mTORC1 signaling pathway and abnormal activation of the 4E-BP1 signaling pathway, resulting in impaired mitochondrial respiratory function, increased ROS production, and increased apoptotic rate. ZSHX recipe can activate mTORC1, then control 4E-BP1, inhibit fatty acid oxidation, protect mitochondrial respiratory function, thereby reducing ROS production, reducing cell apoptosis and promoting ATP synthesis, thus protecting cardiac myocytes from injury. What's more, the protective effect of the ZSHX recipe can also reduce mitochondrial membrane potential, stabilize mitochondrial membrane structure, delay apoptosis, and maintain the cardiac function of myocardial cells after H/R.

## Figures and Tables

**Table 1:** Primer design of mTOR/Raptor/4EBP-1/CPT/ATP.

	Forward (F)	Reverse (R)
mTOR	GTG TGG CAA GAG CGG CAG AC	GT TGG CAG AGG ATG GTC AAG TTG
Raptor	TTC GCA CCG CTC ACT CAT TGT AG	TCG CAC ATC TCC ATT GAC ACT CAC
4EBP1	GGC ACG CTC TTC AGC ACC AC	AGG CGA GTT CCG ACA CTC CAT C
CPT	CAA GTC AAC GGC AGA GCA GAG G	TCA TGG CAG GGC GGT ACA GG
ATP	CCG TCA AAG TGA AAA GCC TAA A	GAC ACT CAT GAT GGT GGA AAA G

## Data Availability

The data used to support the findings of this study are available from the corresponding author upon request.

## Abbreviations

CHD: Coronary heart disease
CABG: Coronary artery bypass graft
PCI: Percutaneous coronary intervention
DMSO: Dimethyl sulfoxide
ATP: Adenosine triphosphate
ROS: Reactive oxygen species
OCR: Oxygen consumption rate
ΔΨ*m*: Mitochondrial membrane potential
mPTP: Mitochondrial permeability transition pore
CRP: C-reactive protein
HCY: Serum homocysteine
HDL: High-density lipoprotein cholesterol.
